# Utility of serum des-gamma-carboxyprothrombin in the diagnosis of hepatocellular carcinoma among Nigerians, a case–control study

**DOI:** 10.1186/s12876-015-0344-9

**Published:** 2015-09-04

**Authors:** Akpakip I. Ette, Dennis A. Ndububa, Olusegun Adekanle, Udeme Ekrikpo

**Affiliations:** 1 Department of Medicine, University of Uyo Teaching Hospital, Uyo, Nigeria; 2 Department of Medicine, Obafemi Awolowo University, Ile-Ife, Nigeria; 3 Department of Medicine, University of Uyo, Uyo, Nigeria

## Abstract

**Background:**

Hepatocellular Carcinoma (HCC) is a common malignancy occurring globally but with a dismal prognosis. Des-gamma-carboxyprothrombin (DCP) has been reported to be more sensitive and specific than Alpha-fetoprotein (AFP) in the diagnosis of HCC among the White population. Its efficacy among the Black population is yet to be established. The aim of this study therefore, was to determine the relative sensitivity and specificity of des-gamma-carboxyprothrombin and alpha-fetoprotein in the diagnosis of hepatocellular carcinoma in a cohort of Nigerian patients presenting at a single referral centre.

**Methods:**

Cross-sectional case–control study was carried out using 62 HCC patients and 57 controls with benign liver diseases including chronic hepatitis and compensated liver cirrhosis. Both DCP and AFP were tested using enzyme immunoassay methods.

**Results:**

Sixty-nine percent of the HCC patients presented with tumour sizes >5 cm while 31 % presented with tumour sizes 3-5 cm. No patient presented with tumour sizes <3 cm. The sensitivity and specificity of DCP were 96.8 % and 98.3 % respectively, based on a Receiver operating characteristic (ROC) curve - derived optimum cut-off level of >140mAU/ml. Similarly, the sensitivity and specificity of AFP were 62.9 % and 93.3 % at an ROC - derived optimum cut-off level of 18mAU/ml. The area under the receiver operating characteristic curve (AUROC) for DCP was 0.99 and was significantly larger than that of AFP which was 0.85 (*p* < 0.001).

**Conclusion:**

In conclusion, the utility of DCP for the diagnosis of HCC among Nigerian patients was higher than that of AFP for large tumours with diameter ≥3 cm.

## Background

Hepatocellular carcinoma is the fifth most common malignancy and the third most common cause of cancer-related deaths world-wide [1]. It is a disease with a dismal prognosis [2]. The poor outcome is related to late detection with more than two thirds diagnosed at the advanced stages [3]. To achieve early diagnosis, attempts have been made at discovering tumour markers. While alpha-fetoprotein (AFP) is the most widely used tumour marker for hepatocellular carcinoma (HCC) surveillance, experience with des-gamma-carboxyprothrombin (DCP) is limited.

Des-gamma-carboxyprothrombin is an abnormal prothrombin released into the blood in the absence of vitamin K or the presence of vitamin K antagonists such as warfarin and is specific to hepatocellular carcinoma [4]. The accuracy of DCP is reduced in prolonged obstructive jaundice, vitamin K deficiency as may occur in severe malnutrition, or the use of vitamin K antagonists such as warfarin [4–7]. It is formed as a result of a defect in post- translational carboxylation of ten glutamic acid residues at the N-terminal end of the prothrombin molecule in malignant cells [8]. It was first detected in the serum of patients with HCC in 1984 by Liebman et al [4]. Thereafter, there have been series of studies comparing the sensitivity and specificity of AFP and DCP in an attempt to aid early diagnosis. Many of the studies have shown DCP to be more sensitive than AFP [9–12]. Some have reported AFP to be more sensitive than DCP [13, 14].

However, further research clarified the discrepancies by showing that the sensitivity of DCP and AFP vary according to tumour sizes. It was found that AFP is more sensitive for the diagnosis of small size tumours (<3 cm) while DCP is more sensitive for larger size tumours (>3 cm) [11, 15–20]. DCP was also found to be the most useful predisposing clinical parameter for the development of portal vein invasion [21].

Most of the studies on DCP have been carried out in the White population, especially in Japan. Few studies have used the Black population. In one of such studies using the Black population, DCP values were found to be significantly higher in cirrhotics and in White patients than in Black patients in America which may indicate a higher sensitivity among Black individuals while the converse may be true for AFP [12]. A study comparing DCP and AFP as markers of HCC in South African Black patients showed that the sensitivity and specificity of DCP were less than those of AFP [13]. To date, there is no report known to the authors regarding the use of DCP in the diagnosis of HCC in Nigeria. The purpose of this study was therefore to compare the sensitivity and specificity of AFP and DCP among Nigerian patients with HCC presenting at the Obafemi Awolowo University Teaching Hospitals Complex, Ile-Ife, in South-Western Nigeria.

## Methods

### Patients

This was a cross-sectional case–control study. Patients were drawn from referrals to the Liver Unit at the Obafemi Awolowo University Teaching Hospitals Complex, Ile-Ife, from April 2011 to March 2012. Sixty-two consecutive patients presenting with untreated primary hepatocellular carcinoma diagnosed using the European Association for the Study of Liver Diseases (EASL) and American Association for the Study of Liver Diseases (AASLD) criteria were enrolled [22, 23]. The controls were 57 patients with benign hepatic diseases which comprised of 34 patients with chronic Hepatitis B infection, one patient with chronic Hepatitis C infection, 21 patients with compensated cirrhosis of the liver, and one patient with Non-alcoholic Fatty Liver Disease . The patients were divided into two broad groups: HCC and non-HCC groups. Ethical clearance was obtained from the hospital ethical committee (Obafemi Awolowo University Teaching Hospital Complex, Ethics and Research Committee clearance number: IRB/IEC/0004553) and informed consent was obtained from all the patients before enrolling them for the study.

A full history and detailed clinical examination was done on all the patients. Laboratory tests carried out included Hepatitis B surface antigen and Hepatitis C antibody tests. Liver function tests including prothrombin time were done by conventional methods. All the cases and controls were subjected to abdominal ultrasound while CT scan was restricted to those shown on ultrasound to have focal lesions in the liver. HCC diameter was measured by ultrasound and/or CT scan. Patients with a history of the use of warfarin or other dicoumarols or total Bilirubin level above 20 mg/dl (340 μmol/l) were excluded from the study. All the patients used for this study were native Nigerians.

Five millilitres of venous blood was collected from all the patients and sera stored at −20 °C until they were analyzed for the tumour markers. The analysis for both tumour markers (DCP and AFP) was carried out at a national research institute: Nigerian Institute for Medical Research, Lagos, Nigeria.

### AFP assay

AFP was tested using commercially available immuno-enzymometric assay kit manufactured by INTECO Diagnostics, UK Ltd., London. The test was carried out as per manufacturer’s instructions. The upper limit of normal for the kits as given by the manufacturer was 10 IU/ml.

### DCP assay

The DCP values were obtained using “Haicatch PIVKA-II” enzyme Immunoassay test kit (Sanko Junyaku co. Ltd., Tokyo, Japan). The tests were carried out as per the manufacturer’s instructions. Briefly, the test involves using anti-DCP monoclonal antibody coated on the inner surface of the micro cup. DCP in the sample is captured and allowed to react with enzyme-labelled anti-human prothrombin antibody. When substrate solution is added to the reaction product, the enzyme reaction develops colour. DCP concentration is determined from the absorbance of the coloured solution. The assay range of the kit was 10–2000 mAU/ml. The upper limit of normal for the kit was given as 40mAU/ml by the manufacturer.

### Tumour- size determination

HCC diameter was measured by ultrasound and/or CT scan and the longest axis was defined as the diameter of the tumour. When HCC tumours were multiple, the largest was measured, and taken as a representative HCC diameter. Based on previously published literature [15, 17] the tumour diameters were grouped as follow:

a <3 cm
b 3-5 cm
c >5 cm.

### Statistical analysis

Data generated from the study was entered into Microsoft Excel and then transferred to STATA 10 software (Statacorp, Texas, USA) for data management and analysis. The baseline socio-demographic and clinical characteristics were reported as mean ± standard deviation computed for normally distributed continuous variables, and median and their corresponding inter-quartile range for non-normal continuous variables.

A comparison of the socio-demographic and clinical characteristics of individuals with or without hepatocellular carcinoma was performed. The student *t*-test was employed to compare the normally continuous variables by HCC status and the Wilcoxon rank sum test employed to compare the non-normal continuous variables. The Pearson’s chi-square was used to find a difference in the proportions of the categorical variables between the two groups. 2x2contingency tables were employed to calculate the sensitivity and specificity of both tests in detecting HCC. Receiver operating characteristic (ROC) curves for both tests were drawn and comparison of the area under the curves (AUROC) for both tests done to establish which test is a better tumour marker for HCC in Nigerians.

In patients with HCC, correlations between AFP and DCP values and various patient characteristics were examined. Non-parametric Spearman rank correlation (international normalised ratio, age, aspartate transaminase (AST), alanine transaminase (ALT), albumin and total bilirubin) was utilized to find the linear relationship between DCP and AFP and other continuous variables listed within the bracket. Significant correlation between two variables was defined at a Spearman rank correlation coefficient of 0.4 or higher and a 2-sided P- value of less than 0.05.

## Results

### Patients characteristics

A total of 119 patients were enrolled in this study, consisting of 62 patients with primary HCC and 57 controls. All the patients used for this study were native Nigerians and all were treatment naive before recruitment into the study.

The baseline demographic and clinical characteristics of both the cases and controls are shown in Table 1. There were 48 males and 14 females among the cases (ratio 3.4:1), while the controls had 38 males and 19 females (ratio 2:1). There was no significant difference in the sex ratio of patients and controls (*p* = 0.19). The mean age of the HCC patients was 46.3 (±15.6) years, while that of the controls was 43.2(±12.2) years. There was no significant difference in the mean ages of patients and controls (*p* = 0.05). The peak age incidence of HCC was in the 35–55 years age- range (table 1). Hepatitis B infection was the most important etiologic factor among the HCC cases (66.1 %) and non- HCC controls (73.7 %).

**Table 1:** Demographic and clinical characteristics of hcc cases and non-hcc controls

	Median (Inter-quartile range) or N (%)		*p-value
Variables	HCC (*n* = 62)	Non-HCC (*n* = 57)	
Age (years)	49.5(35–55)	36(32–51)	*p* = 0.05
Gender M:F	48:14	38:19	*p* = 0.19
AST (u/L)	89(68–112)	12(7–23)	*p* < 0.001
ALT (u/L)	36(22–54)	8(5–16)	*p* < 0.001
Total Bilirubin (μmol/l)	60.3(28–144)	18(11.1-29)	*p* < 0.001
Albumin (g/l)	30(26–35)	36(34–39)	*p* < 0.001
INR	1.19(1–1.4)	1.1(1–1.2)	*p* = 0.22
Positive for HBsAg	41(66.1 %)	42(73.7 %)	*p* = 0.52
Positive for Anti-HCV	0 %)	1(1.8 %)	*p* = 0.30

*P value <0.05 is deemed significant

*AST* aspartate transaminase, *HBsAg* Hepatitis B surface antigen, *ALT* alanine transaminase, *Anti-HCV* Hepatitis C antibody

### Baseline data for AFP and DCP

**Fig. 1:**
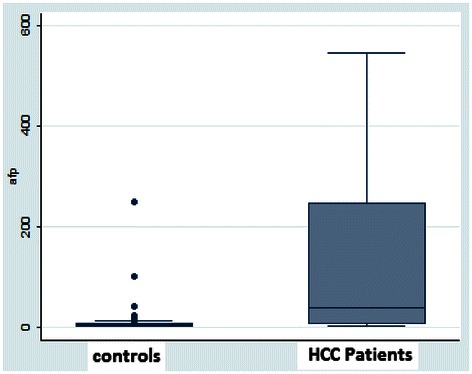
Box Plot for AFP values in both the HCC cases and Non-HCC controls. The Box indicates the 25th and 75th percentile of the data and the middle line indicates the median. A line extends from the minimum to the maximum values excluding outliers that are displayed as separate points

**Fig. 2:**
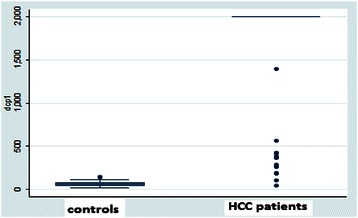
Box Plot for DCP in the HCC cases and non-HCC controls. Outliers are smaller than the lowest quartile minus three times the interquartile range or larger than the highest quartile plus 3 times the interquartile range

Table 2 shows the baseline data for AFP and DCP recorded in mean ± standard deviation. It could be seen from the table, that the serum levels of AFP and DCP were significantly higher in the HCC patients than in the non-HCC controls (*p* < 0.001). The normal values for the DCP kits as given by the manufacturers were 0-40mAU/ml while the normal values for the AFP kits were given as 0–10 IU/ml. The median and the corresponding inter-quartile ranges for AFP were 39(8–245) IU/ml for HCC cases and 4.8(3–7) IU/ml for non-HCC controls respectively (Fig. 1). For DCP, the median and the inter-quartile ranges were 2000(2000–2000) mAU/ml for HCC cases and 58 (40–72) mAU/ml for non-HCC controls respectively (Fig. 2).

**Table 2:** Tumour marker values for hcc cases and non-hcc controls

Variables	Mean ± Standard Deviation		*p-value
	HCC (n = 62)	Non -HCC (n = 57)	
AFP (IU/ml)	122.8 ± 148.2	11.2 ± 34.8	*p* < 0.001
DCP mAU/ml	1657.6 ± 687.1	59.1 ± 27.4	*p* < 0.001

*p-value < 0.05 is deemed significant

### Optimal cut-off values for DCP and AFP in differentiating HCC from benign lvier diseases

**Fig. 3:**
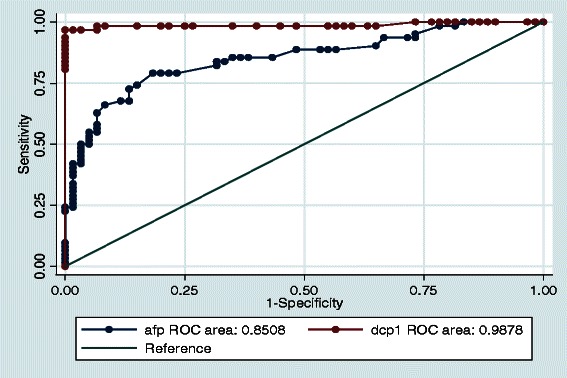
ROC Curves for DCP and AFP in the diagnosis of HCC. The area under the ROC curve (AUROC) for DCP in the diagnosis of HCC is 0.99 (95 % CI = 0.97 – 1.00) compared to the AUROC for AFP in the diagnosis of HCC 0.85 (95 % CI = 0.78 – 0.92), *p* < 0.001

To determine the optimal cut-off value that balances sensitivity and specificity, the ROC curves were plotted for DCP and AFP (Fig. 3). From the ROC curves for DCP, the optimal cut-off value for DCP in differentiating HCC from benign liver diseases stood at 140mAU/ml. Based on this ROC-defined cut-off level, the sensitivity and specificity of DCP were 96.8 and 98.3 % respectively. The AUROC for DCP in the diagnosis of HCC was 0.99 (95 % CI = 0.97-1.00).

From the ROC curve, the optimal cut-off level of AFP was 18 IU/ml. The sensitivity and specificity of AFP in differentiating HCC from benign liver diseases was 62.9 and 93.3 % respectively. The AUROC curve for AFP was 0.85 (95 % CI = 0.78 – 0.92). This was significantly lower than the AUROC for DCP (*p* < 0.001).

### Tumour marker values in relation to tumour profile, disease aetiology and demographics

Table 3 shows the mean ± standard deviation of the tumour markers in relation to the tumour sizes. As could be seen from the table, none of the HCC patients presented with tumour sizes less than 3 cm in diameter. Both DCP and AFP values increased significantly with increasing tumour sizes (*p* = 0.03).

**Table 3:** Values of DCP and AFP in relation to tumour sizes recorded as mean ± standard deviation

	Number of patients (%)	DCP(mAU/ml)	AFP(IU/ml)
HCC	62(100)	1657.4 ± 687.7	122.7 ± 148.3
Tumour Diameter			
< 3 cm	0	0	0
3-5 cm	19(31)	1322.2 ± 858.7	94.9 ± 143.9
> 5 cm	43(69)	1805.5 ± 545.5	135.0 ± 150.2
Non-HCC	57(100)	59.1 ± 27.4	11.2 ± 34.8

Due again to late presentation, only three patients with HCC had solitary-nodule tumours. The Mean ± Standard Deviation (SD) DCP values were 1640 ± 700mAU/ml for single-nodule tumours against 2000 ± 0 mAU/ml for multiple-nodule tumours. No statistically significant correlation was observed with reference to tumour nodularity, probably due to the small number of single- nodule tumours (*p* = 0.4). Extra-hepatic metastasis was noticed in one patient and it was to the chest, presenting as haemorrhagic pleural effusion. The DCP level was >2000 mAU/ml. Further statistical analysis may give misleading results in view of the small number.

As shown in table 4, Hepatitis B virus aetiology was significantly associated with high DCP levels (*p* < 0.001) as well as high AFP values (*p* = 0.03).

**Table 4:** Tumour marker values according to disease aetiology recorded as mean ± standard deviation

Disease Aetiology	Number of patients (%)	DCP(mAU/ml)	AFP(IU/L)
HBV-related HCC	41(66.1 %)	1886.2 ± 410.6	149.0 ± 165.2
Non-HBV-related HCC	21(33.9 %)	1128.3 ± 893.0	59.8 ± 78.4

The DCP levels were also stratified according to gender. The males had significantly higher levels of DCP than females, the Mean ± SD being1832 ± 496.5mAU/ml against 1056 ± 906.8 mAU/ml respectively (*p* < 0.001).

### Sensitivity and specificity of AFP and DCP

The sensitivity and specificity of AFP and DCP at different cut-off points are presented in table 5. It could be seen that as the sensitivity increases, so does the specificity decrease and vice-versa.

**Table 5:** DCP versus AFP in differentiation of patients with HCC from those with benign liver diseases

DCP(mAU/ml)	Sensitivity (%)	Specificity (%)
> 40	100	26.7
> 100	98.4	91.7
> 140	96.8	98.3
> 200	93.6	100
AFP(IU/ml)		
> 10	74.2	85.0
> 18	62.9	93.3
> 20	58.1	93.3
> 100	41.9	96.7
> 00	29.0	98.3

### Correlation between serum AFP, DCP and other patient characteristics

Univariate analysis was carried out using important continuous variables such as international normalised ratio, Age, AST, ALT, Albumin and Total Bilirubin. The univariate analysis identified AST as the only variable that significantly correlated with the DCP values. (Spearman rank correlation coefficient = 0.52 and p < 0.001).It is important to note also that AFP did not correlate with DCP (Spearman rank correlation coefficient = 0.12 and *p* = 0.36).

## Discussion

Hepatocellular carcinoma is a disease with a poor prognosis. Early detection could lead to improved outcome through the application of potentially curable interventions.

To achieve early diagnosis, attempts have been made at discovering tumour markers. Alpha-fetoprotein and Des-gamma-carboxyprothrombin are the most widely used tumour markers but their usefulness in different ethnic groups is still an issue of contention. In this study we compared the usefulness of the two tumour markers in the diagnosis of HCC among Nigerian patients.

Late presentation of HCC patients is commonly reported in sub-Saharan Africa [2, 24–26]. This may explain why none of the HCC patients in this study had tumour nodule sizes < 3 cm at presentation. As noted in this study, the levels of AFP and DCP increased significantly with increasing sizes of the tumour nodules. This finding confirms several reports relating to tumour sizes and AFP and DCP levels [14, 27–29] Durazo et al. [11] in studying 73 patients with HCC noted that the mean DCP value ranged from 7873 mAU/ml for tumour sizes < 3 cm to 12,403 mAU/ml for tumour size > 10 cm. Similarly, the mean AFP values ranged from 599 ng/ml for tumour sizes < 3 cm to 264, 016 for tumour sizes > 10 cm. Nakamura et al. [17] while reporting on 1377 HCC cases gave the median DCP values for tumour sizes < 3 cm, 3-5 cm and > 5 cm as 30mAU/ml, 318 mAU/ml and 6000 mAU/ml respectively. The corresponding AFP values were 25 ng/ml, 48 ng/ml and 556 ng/ml for tumour sizes < 3 cm, 3–5 cm and >5 cm respectively. Non- delineation of tumour sizes led to the discrepancies in earlier reports on the sensitivity and specificity of AFP and DCP. The controversy was largely laid to rest when it was discovered that the sensitivity, specificity and accuracy of AFP and DCP vary according to tumour sizes.

It would be safe to note that no definite inferences or conclusions could be made from our study on the usefulness of Alpha-fetoprotein and Des-gamma- carboxyprothrombin in identifying asymptomatic patients or patients on surveillance as none of the HCC patients presented early (with tumour sizes < 3 cm). Strict surveillance protocol is therefore imperative in the tropics.

From this study, it appears that DCP is more sensitive and specific than AFP in differentiating hepatocellular carcinoma from benign liver diseases among Nigerian patients with tumour sizes ≥ 3 cm. DCP had a sensitivity of 96.8 % and a specificity of 98.3 % at an ROC derived cut-off value of 140 mAU/ml while AFP had a sensitivity of 62.9%and a specificity of 93.3 % at an ROC derived cut-off value of 18 IU/ml. This finding of higher sensitivity and specificity of DCP over AFP conforms to the finding from many reports globally. DCP is known to be more sensitive than AFP in the diagnosis of large size tumours and advanced HCC [11, 15, 20, 29, 30] Sensitivity of 96.8 % is similar to the finding by Wang et al., [18] who reported a sensitivity of 100 % for tumours > 3 cm. In the same vein, Nakamura et al., [17] gave a DCP sensitivity of 95 % for tumour size >5 cm and 77 % for tumour size 3-5 cm. These were higher than AFP sensitivity of 78 % for tumour size > 5 cm and 68 % for tumour size 3-5 cm at a cut-off point of 20 ng/ml. Still in support of the finding in our study of higher sensitivity of DCP for tumour size ≥ 3 cm, Okuda et al., [29] reported a DCP sensitivity of 81.5 % for tumours > 3 cm and 28.6 % for tumour size < 2 cm. The corresponding sensitivity for AFP was 70.4 % and 40 % respectively indicating better sensitivity of AFP for small size tumours. A large multi-centre study in America involving 419 HCC cases and 417 cirrhosis controls showed that for Early Stage HCC, AFP had a better performance/accuracy than DCP but when intermediate- advanced stage HCC (similar to most cases in our study) were compared, then DCP had the better performance/accuracy (AUROC = 0.89for DCP against 0.84 for AFP) [31].

The sensitivity of 96.8 % in our study is much higher than 67.3 % reported in 1988 among South African Black patients [13]. This may be due to the better sensitivity and specificity of the enzyme-linked immunoassay method currently in use, over the coagulase and other similar methods used at that time [15, 16]. It may also be due to the fact that the tumours were not delineated according to their sizes.

In this study of 62 Nigerian patients with HCC, the area under the curve (AUROC) of DCP was significantly larger than that of AFP. This result indicates that DCP is superior to AFP for the diagnosis of large tumours (≥3 cm). Patients with high levels of DCP and low levels of AFP were reported to have large tumours more than 3 cm compared with that of small tumours less than 2 cm, and a somewhat higher frequency of moderately to poorly differentiated HCC compared with that of well differentiated HCC [16, 30]. These results suggest that DCP was closely related to large tumour diameter in comparison to AFP and are in agreement with the results of the ROC analysis performed in our study. The mechanism of this unique characteristic of DCP is not well understood. However, it has been postulated that the aggressive nature of DCP-positive HCC may be due to the fact that DCP stimulates cell proliferation through the Met-Janus Kinase signalling pathway, whereas for vascular endothelial cells, it stimulates cell proliferation and migration through the mitogen – activated protein kinase signalling pathway [32].

Further, alpha-fetoprotein is shown in this study to be less sensitive and of inferior accuracy in the diagnosis of HCC when compared with DCP for patients presenting with tumour size ≥3 cm. The sensitivity of 62.8 % is lower than 83.7 % reported in 1988 in South Africa [13] 66 percent in 1974 in Uganda, [33] and 90 % in 1992 in Zaire [34]. This may partly be due to the fact that some of the studies were done using immuno - diffusion method rather than radioimmunoassay and enzyme immunoassay. It has been found that with these latter (sensitive) methods, a considerable proportion of patients with HCC are not associated with a significant elevation of AFP [35, 36]. This may explain why there is increasingly less reliance on AFP as a diagnostic and surveillance tool in the management of HCC globally [22, 23, 35, 36]. A study published in 1988 using 139 patients over a 4 year period noted that the frequency of AFP negative or low-level AFP hepatocellular carcinoma patients has been increasing gradually [36]. A similar report using 606 patients over a 9- year period arrived at the same conclusion [35].

A previous report from Nigeria gave a positive rate of 64 % at a cut-off level of 200 IU/ml. [37]. The sample size of 14 HCC cases was however too small for any reliable inference to be made from the study. On the other hand a retrospective study from Enugu, Eastern Nigeria reviewed the records of 150 HCC cases, and gave a positive rate of 44.5 %. The cut-off value was however not stated [24].

Lower AFP sensitivity than the figure in our study has been reported in other parts of the world. At a cut-off value of 20 ng/ml a low sensitivity of 63 % for all racial groups and much lower sensitivity of 42.9 % for African Americans has been reported [38] Brunello et al. [39] reported a sensitivity of 43.5 % which is lower than the finding in this study. A multi-centre large scale study involving 1158 patients with HCC, noted a sensitivity of 54 % at a cut-off value of 20 ng/ml. [28]. This rate is also lower than the finding in our study.

Notwithstanding its low sensitivity and accuracy AFP is still the most widely used tumour marker for HCC surveillance and diagnosis but there is the need for discovery of better tumour markers. Univariate analysis in this study showed no correlation between DCP and AFP indicating that the combination of these markers is complementary. DCP is not routinely employed for the diagnosis of HCC in Nigeria principally due to unavailability. With the result of this study, a case may now be made for its use as most cases of HCC seen in Nigeria are in the late stages at presentation.

Hepatitis B viral aetiology was shown in this study to be significantly associated with high DCP and AFP values. A correlation has been found to exist between HBsAg positivity and serum AFP levels in HCC patients. HCC patients who are positive for HBsAg are significantly more likely to be AFP positive and have higher levels of AFP than those who are not, suggesting a relationship between HBV infection and AFP production [27, 33, 40]. Similarly, DCP has also been reported to perform better in those with viral aetiology [31].

Males were found to have significantly higher DCP values than females in our study. This supports a similar report in an earlier study and needs further investigations to explain the underlying mechanism [12].

Univariate analysis in this study showed that a correlation exists between DCP and AST among HCC patients. This is in agreement with the report elsewhere though their report also showed a correlation between AST and AFP which was not seen in our study [9]. A higher AST: ALT ratio has been shown in patients with HCC- compared with those having benign liver diseases likely due to an increase in cytosolic AST [35, 36, 41].

## Conclusion

Our study showed that DCP is superior to AFP in differentiating hepatocellular carcinoma from benign liver diseases in Nigerian patients presenting with large-size tumours (≥3 cm in diameter). A DCP value of >140mAu/ml is associated with a high likelihood that the patient has HCC (sensitivity/specificity ~97 %). Inferences on small size tumours (<3 cm in diameter) could not be made as none of the patients was diagnosed with HCC at an early stage.

Further studies using a larger sample size would be needed to define the optimal cut-off point for DCP in differentiating HCC from benign liver diseases among Nigerians. Research into the role of DCP in the early diagnosis of HCC at potentially curable stages, among Nigerian patients would also be required.

## Abbreviations

HCC: Hepatocellular Carcinoma
DCP: Des-gamma-carboxyprothrombin
AFP: Alpha-fetoprotein
ROC: Receiver operating characteristic
AUROC: Area under the ROC curve
AST: Aspartate Transaminase
ALT: Alanine Transaminase
INR: International normalised ratio
SD: Standard deviation
